# Time course changes in in vivo muscle mechanical function and Ca^2+^ regulation of force following experimentally induced gradual ovarian failure in mice

**DOI:** 10.1113/EP091735

**Published:** 2024-03-18

**Authors:** Avery Hinks, Benjamin E. Dalton, Parastoo Mashouri, Luke D. Flewwelling, William Glen Pyle, Arthur J. Cheng, Geoffrey A. Power

**Affiliations:** ^1^ Department of Human Health and Nutritional Sciences, College of Biological Sciences University of Guelph Guelph Ontario Canada; ^2^ Muscle Health Research Centre, School of Kinesiology and Health Sciences, Faculty of Health York University Toronto Canada; ^3^ IMPART Team Canada, Dalhousie Medicine Dalhousie University Saint John New Brunswick Canada

**Keywords:** menopause, muscle, ovarian hormones, ovaries, perimenopause, power, VCD

## Abstract

The abrupt cessation of ovarian hormone release is associated with declines in muscle contractile function, yet the impact of gradual ovarian failure on muscle contractility across peri‐, early‐ and late‐stage menopause remains unclear. In this study, a 4‐vinylcyclohexene diepoxide (VCD)‐induced ovarian failure mouse model was used to examine time course changes in muscle mechanical function. Plantar flexors of female mice (VCD: *n* = 10; CON: *n* = 8) were assessed at 40 (early perimenopause), 80 (late perimenopause), 120 (menopause onset) and 176 (late menopause) days post‐initial VCD injection. A torque–frequency relationship was established across a range of frequencies (10–200 Hz). Isotonic dynamic contractions were elicited against relative loads (10–80% maximal isometric torque) to determine the torque–velocity–power relationship. Mice then performed a fatigue task using intermittent 100 Hz isometric contractions until torque dropped by 60%. Recovery of twitch, 10 Hz and 100 Hz torque were tracked for 10 min post‐task failure. Additionally, intact muscle fibres from the flexor digitorum brevis underwent a fatigue task (50 repetitions at 70 Hz), and 10 and 100 Hz tetanic [Ca^2+^] were monitored for 10 min afterward. VCD mice exhibited 16% lower twitch torque than controls across all time points. Apart from twitch torque, 10 Hz torque and 10 Hz tetanic [Ca^2+^], where VCD showed greater values relative to pre‐fatigue during recovery, no significant differences were observed between control and VCD mice during recovery. These results indicate that gradual ovarian failure has minimal detriments to *in viv*o muscle mechanical function, with minor alterations observed primarily for low‐frequency stimulation during recovery from fatigue.

## Abstract (highlights)

- **What is the central question of this study?**
  In a mouse model of gradual ovarian failure (4‐vinylcyclohexene diepoxide, VCD), how does muscle mechanical function change during the perimenopausal transition into early and late menopause?
- **What is the main finding and its importance?**
  VCD and control mice experienced similar time course changes in mechanical function, and exhibited mostly similar impairments in function during a fatigue task and throughout recovery. These findings indicate there is minimal impact of gradual ovarian failure on joint level muscle mechanical function.

## 1 INTRODUCTION

Over the past several decades, the beneficial effects of ovarian hormones on physiological function in females has gained interest, particularly in terms of muscle function (Enns & Tiidus, 2010; Pellegrino et al., 2022). Importantly, age‐matched males and females have similar reductions in specific force (i.e., force per muscle cross‐sectional area) up until the fifth decade of life (i.e., after the onset of menopause), whereafter females not on hormone replacement therapy exhibit a more dramatic decline in muscle function than males (Phillips et al., 1993). These findings indicate the importance of ovarian hormones in maintaining muscle contractile function in older females, and the deleterious effects of postmenopausal reductions in circulating ovarian hormones on muscle contractility.

To study the effects of a menopausal reduction in the levels of ovarian hormones on muscle contractile function, an ovariectomy (OVX) rodent model involving the surgical removal of the ovaries is commonly used (Greising et al., 2011; Moran et al., 2007, 2006). Extensor digitorum longus (EDL) and soleus (SOL) muscles from OVX mice (∼60 days post‐ovariectomy) produce ∼20% lower specific force than those from sham mice (Moran et al., 2006). These reductions in specific force may be attributed to changes in Ca^2+^ regulation of force‐ or cross‐bridge‐based impairments. Cross‐bridge‐based impairments are likely as it has been shown that SOL fibre Ca^2+^ sensitivity remains unchanged 10–14 weeks following OVX (Wattanapermpool & Reiser, 1999). Therefore, there is a reduction in muscle quality and ability to produce force following complete removal of ovarian hormones (Moran et al., 2006). Convincingly, administration of exogenous 17β‐estradiol, one of the primary estrogens produced and released by the ovaries, to OVX mice attenuates the impairments in force generation in OVX mice (Moran et al., 2007), further supporting the crucial role of 17β‐estradiol in muscle force production.

Unlike force production, maximum shortening velocity of intact EDL (Moran et al., 2006) and SOL muscles (Greising et al., 2011) and SOL single muscle fibres (Wattanapermpool & Reiser, 1999) remains unaffected, or even improves (Moran et al., 2006), following OVX as compared with controls. Power (the product of force and velocity), however, is still affected by removal of ovarian hormones. Using a skeletal muscle α‐estrogen receptor‐gene knockout (skmERαKO) mouse model, Cabelka et al. (2019) performed in vivo assessments of plantar flexor function and found that skmERαKO mice produced 7–8% less force and 11–12% less power compared to controls during 100 and 200° s^−1^ isokinetic contractions, and were more fatigable than controls, indicating that 17β‐estradiol is vital for optimal muscle contractile function (Cabelka et al., 2019). Thus, estrogen receptors in skeletal muscle are a primary mediator for 17β‐estradiol's effect on muscle function (Collins et al., 2018) and it appears that 17β‐estradiol is necessary to maintain skeletal muscle power, primarily due to its protective effects on force production while shortening velocity appears to be less dependent on estrogen availability.

Although the OVX model has provided insight into the role of ovarian hormones on muscle contractile function, that model does not mimic the prolonged and complex hormonal transition that includes retention in androgen production by the ovaries (Fernandes et al., 2019; Mark‐Kappeler et al., 2011). Recent development of the occupational chemical 4‐vinylcyclohexene diepoxide (VCD) has offered an experimental design that keeps the ovaries intact while gradually reducing the amount of circulating ovarian hormones (Brooks et al., 2016; Fernandes et al., 2019; Mark‐Kappeler et al., 2011; Springer et al., 1996). VCD targets the primary and primordial ovarian follicles, leading to accelerated atresia, resulting in a rodent model that can be used to mimic the natural trajectory of human menopause (Mark‐Kappeler et al., 2011). Recent work from our lab showed that SOL single fibres from VCD mice produced 33% more force and had greater Ca^2+^ sensitivity at higher Ca^2+^ concentrations compared to controls, with no differences observed for the EDL (Mashouri et al., 2023). It should be noted that these fibres were taken from mice at the onset of complete ovarian failure (i.e., ‘menopause’), which would correspond with the day of ovariectomy in the OVX model, so the deleterious effects of ovarian hormone loss may be minimal. In late‐stage menopause, we also showed that SOL and EDL single fibres of VCD mice produced 31% lower force and 32% lower power than controls (Hubbard et al., 2023). These findings emphasize the importance of assessing muscle contractile performance across the development of both perimenopause and menopause. Most of the studies mentioned above utilized in vitro methods, which requires the animal to be killed, making longitudinal time course observations impossible. An in vivo model, which we use in the current study, would instead allow for time course assessments of muscle function throughout the progression of ovarian failure.

The purpose of this study was to use an in vivo mouse model of VCD‐induced gradual ovarian failure to investigate neuromuscular function of the plantar flexors throughout the peri‐menopause stage to early‐ and late‐stage menopause. We hypothesized that VCD‐induced ovarian failure would cause a downward shift in the torque–frequency curve (i.e., gradual reduction in circulating estrogens would lead to muscle weakness), and a reduction in power production, primarily owing to decrements in torque production while shortening velocity would be maintained. As well, fatiguability would be elevated in the VCD group.

## 2 METHODS

### 2.1 Ethical approval

All procedures were conducted in accordance with the guidelines set by the Animal Care and Use Committee of the University of Guelph (AUP 4714) and the Canadian Council on Animal Care.

### 2.2 Baseline measures and VCD administration

Eighteen CD1 female mice (VCD: *n* = 10, control (CON): *n* = 8) aged 78–105 days were purchased from Charles River Laboratories (St Constant, QC, Canada). Mice were housed four per cage on a 12 h light–dark cycle. Food and water were provided ad libitum. Animals were allowed to acclimatize to new housing for at least 1 week prior to the initiation of VCD treatment.

**FIGURE 1:**
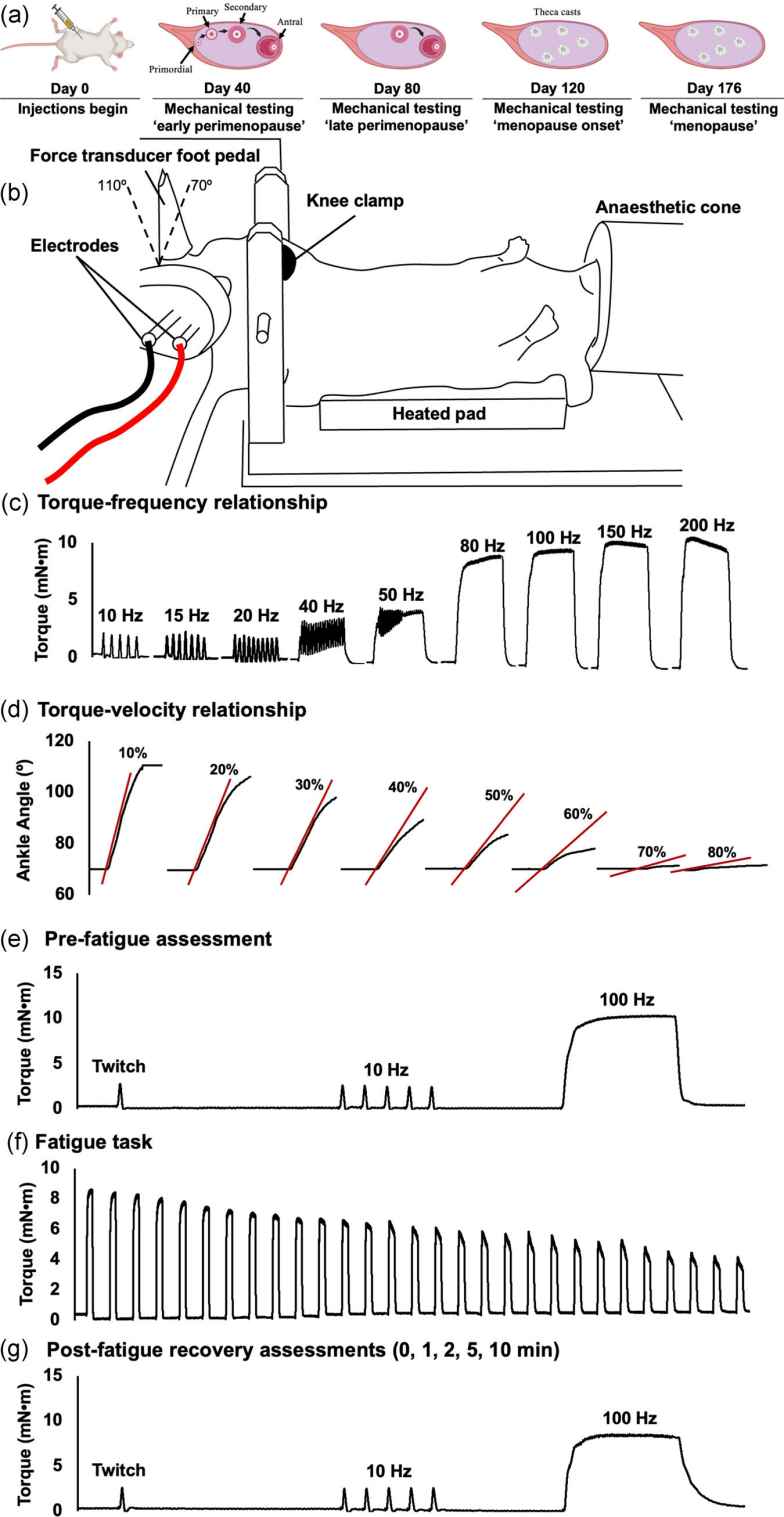
Experimental set‐up and timeline. (a) Mice were injected with 4‐vinylcyclohexene diepoxide (VCD). VCD targets the primordial and primary ovarian follicles, leading to accelerated atresia (day 40–80). Ovarian failure (menopause) is achieved by 120 days following VCD injection, leaving only androgen‐producing tissue in the ovaries. (b) The mouse's left foot was secured to a foot pedal and electrically stimulated transcutaneously to evoke plantar flexion contractions, with the knee clamped at 90°. The ankle went through a 70° to 110° range of motion. (c) Following determination of the optimal current for 100 Hz stimulation, the torque–frequency relationship was constructed in order from 10 to 200 Hz. (d) 100 Hz isotonic contractions from 70° to 110° were performed at load clamps of 10% to 80% of maximum isometric torque at 70°, in a randomized order. (e) A pre‐fatigue assessment was performed that included a twitch stimulation, a 10 Hz stimulation, then a 100 Hz stimulation. (f) The fatigue task was then performed, consisting of repeated 100 Hz stimulations until torque dropped by 60%. (g) The pre‐fatigue assessment was repeated immediately and 1, 2, 5 and 10 min following termination of the fatigue task. Red lines represent the maximum derivative of the position‐time trace (i.e., the measurement of isotonic shortening velocity).

Following acclimatization, mice assigned to the VCD group were injected with 160 mg/kg/day of VCD for 15 days to induce ovarian failure (Brooks et al., 2016; Fernandes et al., 2019; Mashouri et al., 2023). To investigate the effects of gradual ovarian failure on neuromuscular function, mechanical testing was performed around days 40 (40 ± 3 days), 80 (82 ± 2 days), 120 (120 ± 3 days) and 176 (176 ± 3 days) (D40, D80, D120 and D176, respectively) after the onset of dosing (see Figure 1a for a complete timeline). Visual inspection of the vaginal opening was performed every day starting 5 days prior to each testing time point as described previously (Byers et al., 2012; Champlin et al., 1973). The pro‐estrus phase is characterized by swollen, moist, pink tissue (Byers et al., 2012; Champlin et al., 1973). At time points prior to the onset of menopause (i.e., D40, D80), mechanical testing was performed during the pro‐estrus phase due to this phase of the estrous cycle being associated with the greatest 17β‐estradiol concentrations. In diestrus, the vaginal opening is small and closed with no tissue swelling (Byers et al., 2012; Champlin et al., 1973). We ensured that mice remained in the diestrus phase for five consecutive days prior to D120 to indicate that ovarian failure did in fact occur (Brooks et al., 2016; Mashouri et al., 2023). Body mass was measured during each mechanical testing session.

### 2.3 in vivo set‐up

Mice were anaesthetized via inhalation of 3% (O_2_ flow rate of 3 l/min) isoflurane until the desired plane of anaesthesia was achieved (Adams & Pacharinsak, 2015), then transferred to a 37°C heated platform (Aurora Scientific, Aurora, Ontario, Canada), and placed in a supine position (Figure 1b). An ocular lubricant was then applied to the surface of the eyes, and the head was placed in a custom‐built anaesthetic cone. Anaesthesia was maintained throughout testing via inhalation of 1.5% isoflurane with an O_2_ flow rate of 1.5 l/min. The right limb was shaved, and their foot was secured to a footplate attached to the shaft of a motor‐driven dynamometer (300C‐LR; Aurora Scientific). The hip and knee were positioned at 90° (full extension = 180°). Custom‐made transcutaneous electrodes were connected to a stimulator (701C; Aurora Scientific) and placed at the popliteal fossa and Achilles tendon to elicit contractions of the plantar flexors (Figure 1b) (Gerlinger‐Romero et al., 2019).

### 2.4 Electrical stimulation

Stimulation current and electrode placement were optimized during 5–15 isometric tetanic contractions (500 ms train of 0.1 ms pulses at 100 Hz) (Cabelka et al., 2019). Stimulation current was initially set at 20 mA and was progressively increased in 5 mA increments until no further increase in torque was observed. This stimulation current was used throughout the remainder of the testing session.

### 2.5 Torque–frequency relationship

Torque was measured during nine 500‐ms isometric contractions across a range of stimulation frequencies (10, 15, 20, 40, 50, 80, 100, 150 and 200 Hz), each separated by 2 min of rest (Figure 1c). Each contraction was elicited with the ankle at 70° (90° = neutral ankle position) because during piloting, we found that this ankle angle produced the highest active plantar flexion torque among the angles available with the Aurora system (70° to 110°). Passive torque was determined following the initial footplate movement to 70° then 2 s of rest to control for stress‐relaxation, and subsequently subtracted from total torque to determine active torque. For tetanic contractions, peak isometric torque was defined as the highest point along the torque–time curve. To minimize the confounding effects of potentiation, contractions were elicited in order of increasing stimulation frequency (Warren et al., 2004). The torque–frequency relationship was then fitted to the data using the following equation:

$$f\;\left(x\right)=\min+\;\left(\max-\;\min\right)/\left(1+{\left[x/F_{50}\;\right]}^{n}\right).$$

where *x* is the stimulation frequency, min (i.e., 10 Hz) and max (i.e., peak tetanic) are the estimated respective torques (normalized to maximum), *F*_50_ is the stimulation frequency at which half‐maximum torque is achieved, and *n* is the coefficient describing the slope of the steepest portion of the torque–frequency curve.

Additionally, rate of torque development (RTD) was calculated during the 200 Hz isometric tetanic contractions as:

$$\mathrm{RTD}\;=\frac{\Delta \;\mathrm{torque}\;}{\Delta \;\mathrm{time}\;}\;$$

Contraction onset was defined as the point that torque exceeded 3 standard deviations of baseline torque (Maffiuletti et al., 2016). RTD was measured from contraction onset until torque exceeded 95% of maximum isometric peak torque. Each contraction was visually inspected to ensure proper determination of contraction onset and manually adjusted if necessary.

### 2.6 Torque–angular velocity–power relationship

To assess the torque–velocity–power relationship, the foot was passively moved to an ankle angle of 70° and the muscle was stimulated for 500 ms at 100 Hz (Figure 1d). During piloting, 100 Hz produced the highest torque values, with anything beyond 100 Hz inducing antagonist activation such that total plantar flexion torque decreased; therefore, even though 200 Hz yielded the highest tetanic torque values in the final data set, 100 Hz was used for isotonic contractions. Isotonic contractions were performed against a range of relative loads (10%, 20%, 30%, 40%, 50%, 60%, 70% and 80% peak torque) in a random order, each separated by 2 min of rest. The end range of motion was set to 110°, with the total range of motion corresponding closely to that of the ankle during the stance phase of rodent voluntary ambulation (i.e., from 72 to 111°) (Varejão et al., 2002). Angular velocity against each relative load was determined as the maximum derivative of the position–time trace. Torque and angular velocity data were fitted to the Hill equation (Alcazar et al., 2017):

$$\left(P+a\right)\;\left(V+b\right)=\left(P_{o}+a\right)\;b$$

where *P* is torque, *V* is angular velocity, *P*_O_ is peak isometric torque and *a* and *b* are constants with dimensions of torque and angular velocity, respectively (Hill & Sec, 1938). The ratio *a*/*P*_O_ denotes the curvature of the torque–velocity relationship. Maximum shortening velocity (*V*_max_) was determined as the resultant y‐intercept (i.e., torque = 0). A torque‐power curve was established by multiplying the torque and velocity values obtained from the fitted torque–velocity curve and peak power was then determined.

### 2.7 Fatigue and recovery

To assess fatiguability, repeated 500 ms isometric contractions were elicited using 100 Hz stimulations every 2 s (Figure 1f). The fatigue task was terminated once torque dropped by 60% compared to the pre‐fatigue maximum and the number of contractions were counted.

Recovery from fatigue was tracked at time points prior to the fatigue task, then immediately, and at 1, 2, 3, 4, 5 and 10 min following termination of the fatigue task (Figure 1e,g). At each time point, we assessed twitch torque, twitch half‐relaxation time (HRT), twitch RTD, 10 Hz torque, 100 Hz torque, the 10:100 Hz torque ratio and tetanic RTD.

### 2.8 Enzymatic dissociation of intact muscle fibres

Following the final mechanical testing session (i.e., D176), animals were killed via CO_2_ asphyxiation and cervical dislocation. The hindlimbs were removed, and flexor digitorum brevis (FDB) muscles were isolated and cleaned of tendons, connective tissue and blood vessels. Muscles were then transferred into a multi‐welled plate (cat. no. 83.3921.005, Sarstedt, Nümbrecht, Germany) with 3 mL of a solution containing: Dulbecco's modified Eagle's medium (DMEM)/nutrient mixture F‐12, pH 7.4 (cat. no. 12800017, Thermo Fisher Scientific, Waltham, MA, USA); 20% heat‐inactivated fetal bovine serum (FBS; cat. no. F2442, Millipore‐Sigma, Darmstadt, Germany); antibiotic antimycotic solution (6 µL/mL; P4333 Sigma‐Aldrich, St Louis, MO, USA); 0.6% collagenase Type I (SCR103, EMD Millipore Corp., Burlington, MA, USA), and 0.01% sodium bicarbonate. The plate was incubated at 37°C in a water‐saturated incubator (Fisherbrand Isotemp, Fisher Scientific, Pittsburgh, PA, USA) for 2–3 h and then transferred to 1 mL of fresh DMEM/F‐12 with 20% FBS and antibiotic antimycotic solution. Muscles were then gently triturated to dissociate the intact muscle fibres without creating air bubbles. Glass‐bottomed Petri dishes with a diameter of 35 mm (cat. no. P35G‐1.5‐14‐C, MatTek, Ashland, MA, USA) were placed on a sterilized incubator tray, coated with laminin (cat. no. CB‐40232, Thermo Fisher Scientific) and left at room temperature for 2 h before being washed with phosphate‐buffered saline (cat. no. 10010023, Thermo Fisher Scientific). Petri dishes were loaded with 200 µL of the muscle fibre suspension and left for 15 min to allow fibres to attach to the bottom of the Petri dish. Finally, 3 mL of DMEM/F‐12 with 20% FBS and antibiotic antimycotic solution was added, and the cells were incubated in a water‐saturated incubator at 37°C overnight.

### 2.9 Dish preparation

Dishes were removed from the incubator, and excess DMEM/F‐12 with 20% FBS and antibiotic antimycotic solution was removed from the glass‐bottomed Petri dish with 1.5 mL of the solution remaining in the dish. Fifteen microlitres of a solution containing 5 µM of Fura‐2 AM dye (cat. no. ab120873, Abcam, Waltham, MA, USA), 70 µM dimethyl sulfoxide (cat. no. 1003427034, Sigma‐Aldrich), and 5 µL of 8 mM Pluronic (cat. no. 102441509, Sigma‐Aldrich) was added into the dish and the dish was gently swirled so the dye was evenly distributed over the fibres before being incubated at 37°C for 30 min. Once finished, the dishes were moved in the dark onto an inverted microscope (CK40, Olympus Life Science, Tokyo, Japan) and continuously superfused via an analog pump (Reglo, Ismatec, Glattburg, Switzerland) with room temperature (∼25°C) experimental Tyrode solution (mM): 121 NaCl, 5.0 KCl, 1.8 CaCl_2_, 0.5 MgCl_2_, 0.4 NaH_2_PO_4_, 24 NaHCO_3_, 0.1 EDTA, 5.5 glucose, and ∼0.2% heat‐inactivated newborn calf serum (cat. no. 26010074, Thermo Fisher Scientific), bubbled with 95% O_2_–5% CO_2_ giving the bath a pH of ∼7.4 while remaining in the dark.

### 2.10 Intact fibre stimulation protocol

Isolated FDB fibres were electrically stimulated in a custom‐made chamber (holding two parallel platinum electrodes positioned on each side of the light path, 1 cm apart) connected to an isolated high‐power stimulator (Model 4100, A‐M Systems, Sequim, WA, USA). The stimuli and repetitive tetanus frequency were controlled by AMS 4100 software (A‐M Systems) on a Windows PC. Images were acquired using an inverted microscope equipped with a ×40 oil immersion objective. Excitation wavelengths of 360 and 380 nm for fura2 were emitted using an illuminator (DeltaRAM X, Horiba Scientific, Kyoto, Japan) with emissions recorded at the 510 nm wavelength using a digital camera (pco.panda 4.2, Excelitas PCO GmbH, Kelheim, Germany) (Westerblad & Allen, 1991). Image acquisition was recorded using EasyRatioPro 3 software (Horiba Scientific). Background subtraction was done by collecting the fluorescence from an area away from any fibres and subtracting that value from those recorded during contractions.

Muscle fibre(s) were stimulated at 30 V with 500 ms pulse width at frequencies identical to the in vivo torque–frequency assessment (10–200 Hz) with each separated by 2 min of rest. Thereafter, to assess maximal Ca^2+^ release from the sarcoplasmic reticulum (SR), the fibres were then exposed to 5 mM caffeine for ∼2 min and stimulated with a 200 Hz tetanus, and then a washout of caffeine with normal Tyrode solution for 5 min (Westerblad & Allen, 1991). All experiments were performed at room temperature (∼25°C).

Fatigue was induced with repeated 70 Hz tetani of 500 ms duration given every 2 s for a total of 50 tetani. To determine the extent of prolonged low‐frequency force depression, fibres were stimulated with a 10 Hz tetanus and a 100 Hz tetanus immediately post‐fatigue, and at 1, 2, 5 and 10 min after the fatigue protocol. All experiments were performed at room temperature (∼25°C).

### 2.11 Statistical analysis

Three‐way repeated measures analysis of variance (ANOVA) was used to assess changes in the torque–frequency relationship across time and between VCD and control, with time (D40, D80, D120 and D176) and frequency (10, 15, 20, 40, 50, 80, 100, 150 and 200 Hz) as within‐subject factors and group (control, VCD) as a between‐subject factor. Three‐way repeated measures ANOVA was also used to assess changes in the torque–angular velocity relationship across time and between VCD and control, with time (D40, D80, D120 and D176) and load (10%, 20%, 30%, 40%, 50%, 60%, 70% and 80% of maximum) as within‐subject factors and group (control, VCD) as a between‐subject factor. Two‐way repeated measures ANOVA (time × group) was used to compare 100 Hz torque, tetanic RTD, twitch torque, twitch HRT, twitch RTD, *F*_50_, the *n* slope coefficient of the torque–frequency relationship, curvature of the torque–angular velocity relationship (*a*/*P*_O_), *V*_max_, peak power, torque and velocity at peak power, and repetitions to failure across time and between control and VCD. To assess recovery of twitch torque, twitch HRT, twitch RTD, 10 Hz torque, 100 Hz torque, the 10:100 Hz torque ratio and te3tanic RTD following the fatigue task, we used three‐way repeated measures ANOVAs on values normalized to pre‐fatigue with time (D40, D80, D120 and D176) and recovery (pre‐fatigue, 0, 1, 2, 5 and 10 min) as within‐subject factors and group (control, VCD) as a between‐subject factor. To assess differences in the tetanic [Ca^2+^]–frequency relationship between control and VCD, we used a two‐way repeated measures ANOVA with frequency (10, 15, 20, 40, 50, 80, 100, 150 and 200 Hz, and 200 Hz with caffeine) as a within‐subject factor and group (control, VCD) as a between‐subject factor. To assess differences in recovery of 10 Hz tetanic [Ca^2+^], 100 Hz tetanic [Ca^2+^] and the 10:100 Hz tetanic [Ca^2+^] ratio between control and VCD following the fatigue task, we used a two‐way repeated measures ANOVA on values normalized to pre‐fatigue with recovery (pre‐fatigue, 0, 1, 2, 5 and 10 min) as a within‐subject factor and group (control, VCD) as a between‐subject factor. Where significant effects and interactions were detected, effect sizes were reported as the partial eta squared ($\eta_{p}^{2}$). All statistical analyses were performed using SPSS version 28 (IBM Corp., Armonk, NY, USA) at an α level of *P *< 0.05.

## 3 RESULTS

### 3.1 Differences in peak twitch torque, twitch half‐relaxation time and twitch rate of torque development between control and VCD and across time

**FIGURE 2:**
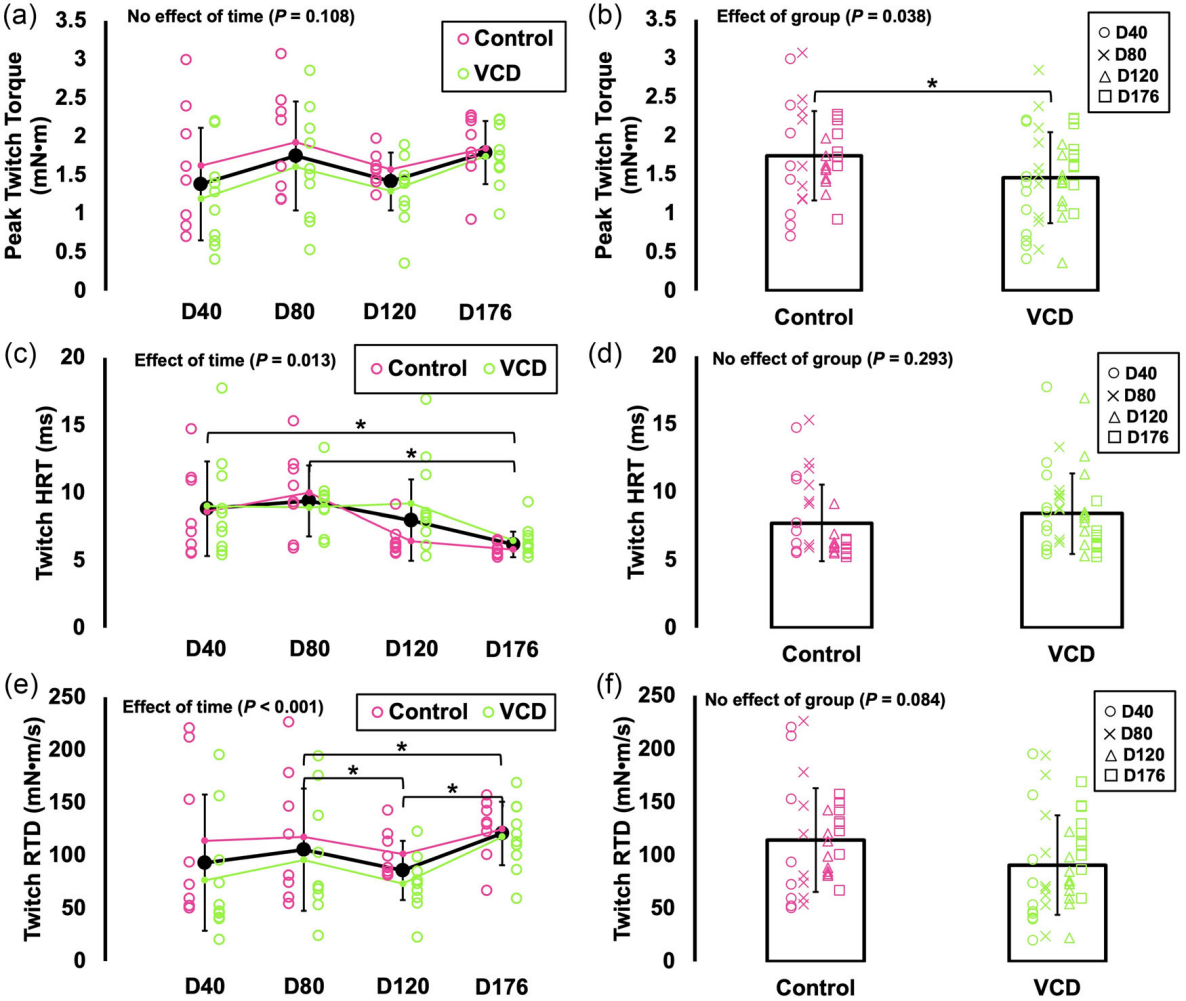
Differences in peak twitch torque (a, b), twitch half‐relaxation time (HRT) (c, d), and twitch rate of torque development (RTD) (e, f) across time and between control (*n* = 8) and VCD (*n* = 10). Effects of time (control and VCD grouped) and effects of group (all time points grouped) are displayed because there was no time × group interaction for any measure. *Difference between indicated points (*P* < 0.05). Data are presented as means ± standard deviation.

For peak twitch torque, there was no effect of time (*F*(2.474, 39.580) = 2.250, *P* = 0.108), indicating no differences across time points (Figure 2a). However, there was an effect of group (*F*(1, 16) = 5.126, *P* = 0.038, η_p_^2^ = 0.038), with VCD mice producing 16% lower peak twitch torque than control mice (Figure 2b).

For twitch HRT, there was an effect of time (*F*(1.851, 29.612) = 5.258, *P* = 0.013, η_p_^2^ = 0.247) such that twitch HRT at day 176 was 30–34% faster than at days 40 (*P* = 0.048) and 80 (*P* < 0.001) (Figure 2c). There was no effect of group on twitch HRT (*F*(1, 16) = 1.180, *P* = 0.293), indicating no difference between control and VCD (Figure 2d).

For twitch RTD, there was an effect of time (*F*(2.455, 39.287) = *P* < 0.001, η_p_^2^ = 0.345). Specifically, twitch RTD at day 80 was 23% greater than at day 120 (*P* = 0.046), and twitch RTD at day 176 was 15% and 41% greater than at day 80 (*P* < 0.001) and 120, respectively (*P* = 0.037) (Figure 2e). There was no effect of group on twitch RTD (*F*(1, 16) = 3.398, *P* = 0.084), indicating no difference between control and VCD (Figure 2f).

### 3.2 Differences in 200 Hz torque and rate of torque development between control and VCD and across time

**FIGURE 3:**
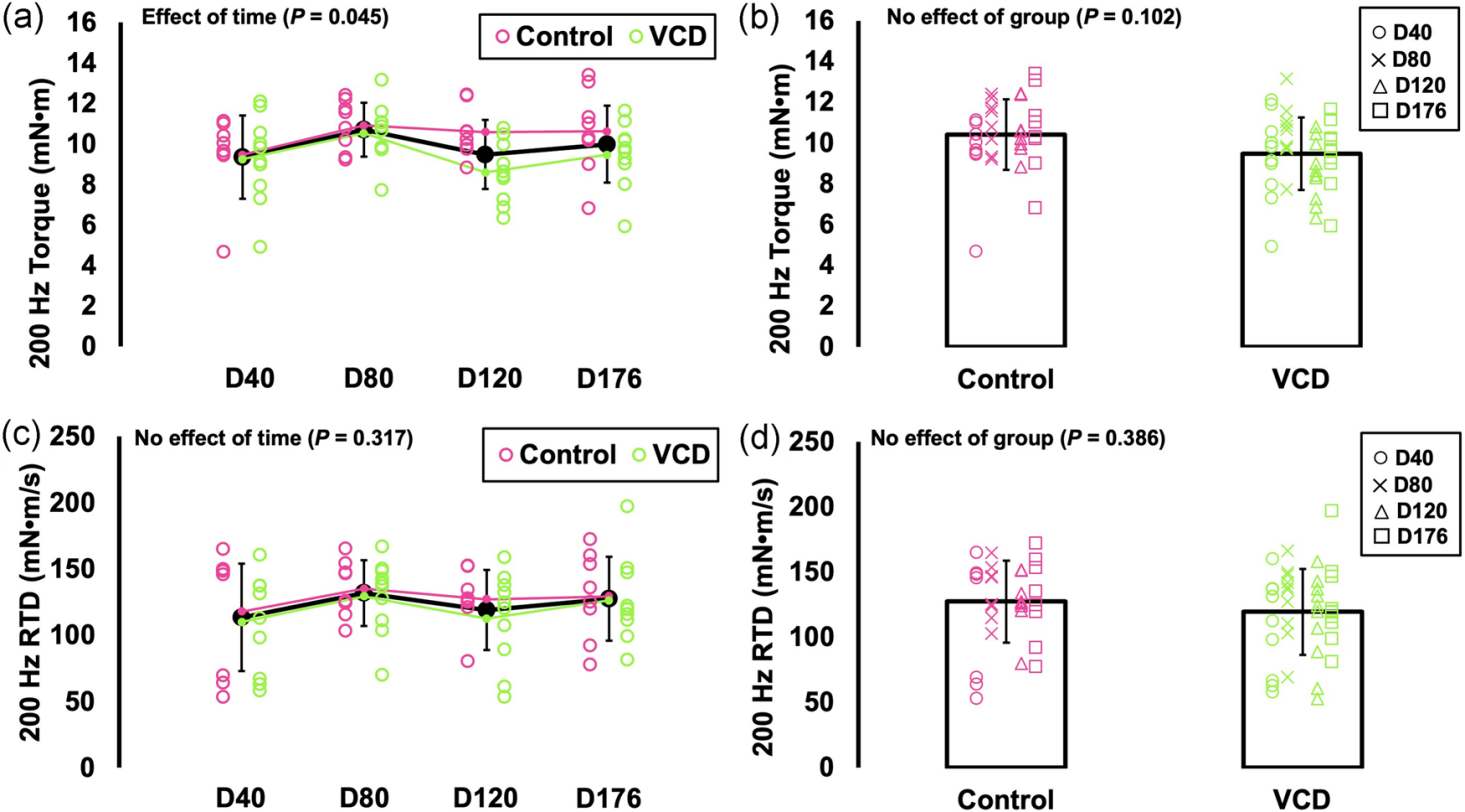
Differences in 200 Hz torque (a, b) and tetanus rate of torque development (RTD) (c, d) across time (day 40, 80, 120 and 176) and between control (*n* = 8) and VCD (*n* = 10). Effects of time (control and VCD grouped) and effects of group (all time points grouped) are displayed because there was no time × group interaction for any measure. *Difference between indicated points (*P* < 0.05). Data are presented as means ± standard deviation.

For 200 Hz torque, there was an effect of time (*F*(2.746, 43.940) = 2.994, *P* = 0.045, η_p_^2^ = 0.158) (Figure 3a). However, pairwise comparisons did not reveal any differences between specific time points, therefore, the effect of time on 200 Hz torque seemed to be small. There was no effect of group on 200 Hz torque (*F*(1, 16) = 3.010, *P* = 0.102), indicating no difference between control and VCD (Figure 3b).

For tetanic RTD, there were no effects of time (*F*(2.199, 35.178) = 1.198, *P* = 0.317) or group (*F*(1, 16) = 0.793, *P* = 0.386), indicating no differences across time points or between control and VCD (Figure 3c,d).

### 3.3 Differences in the torque–frequency relationship between control and VCD and across time

**FIGURE 4:**
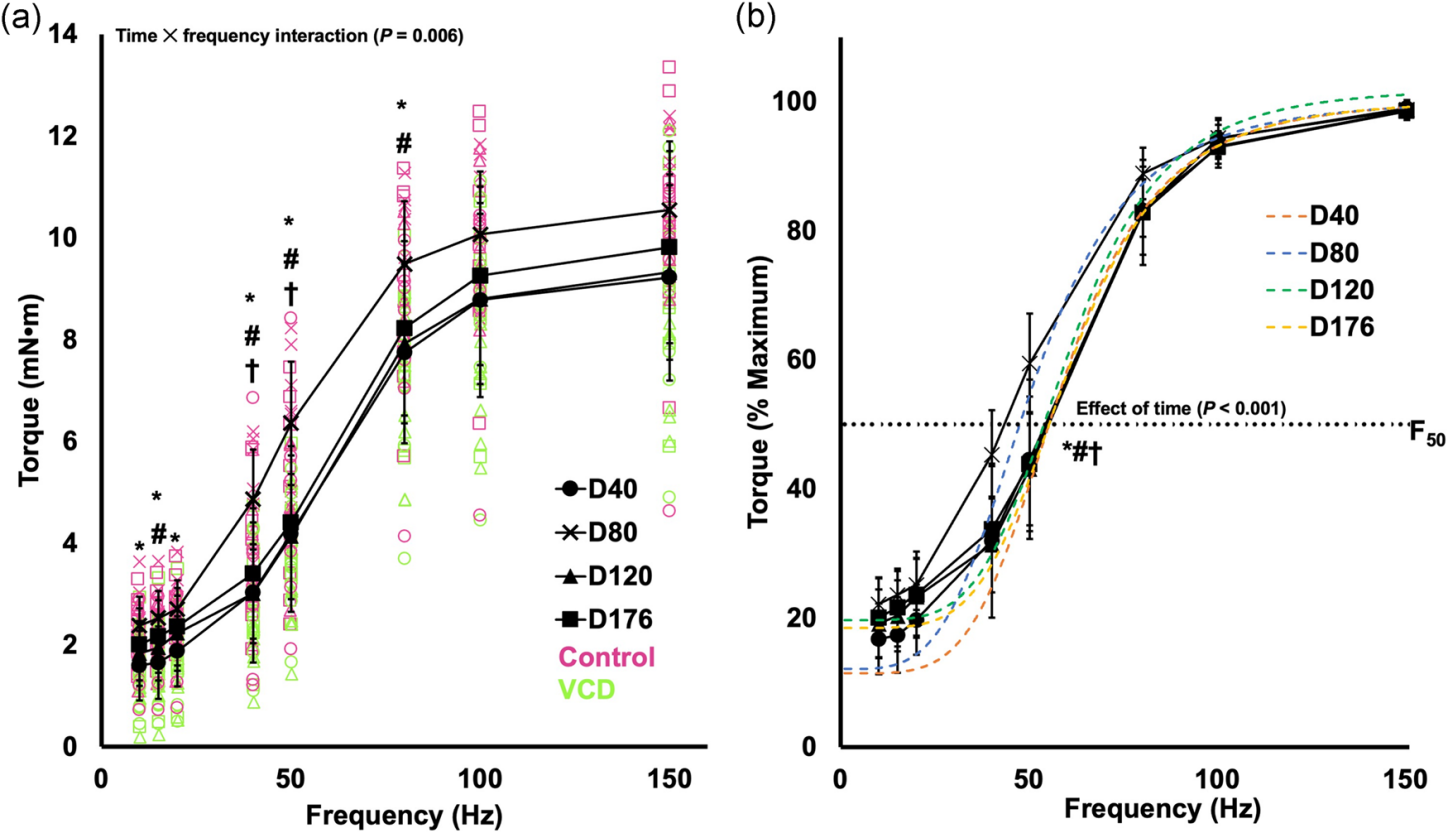
(a) The absolute torque–frequency relationship at each time point (day 40, 80, 120 and 176) with control (*n* = 8) and VCD (*n* = 10) grouped, as there was no effect of group. *Difference between day 40 and 80 (*P* < 0.05). #Difference between day 80 and 120 (*P* < 0.05). †Difference between day 80 and 176 (*P* < 0.05). Data are presented as means ± standard deviation. (b) The normalized torque–frequency relationship along with the fitted curves. The dotted line shows the frequency at which 50% of maximum torque was achieved (*F*_50_). *Difference between day 40 and 80 (*P* < 0.05). #Difference between day 80 and 120 (*P* < 0.05). †Difference between day 80 and 176 (*P* < 0.05). Data are presented as means ± standard deviation.

There was a time × frequency interaction for active torque (*F*(4.950, 79.197) = 3.568, *P* = 0.006, η_p_^2^ = 0.182), indicating the torque–frequency relationship differed across time points. Torque tended to increase with increasing stimulation frequency (*P* < 0.001–0.017). At frequencies of 10–80 Hz, torque was 23–60% greater at day 80 than day 40 (*P* = 0.002–0.011) (Figure 4a). At 15, 40, 50 and 80 Hz, torque was ∼16%–38% lower at day 120 than day 80 (*P* < 0.001–0.049) (Figure 4a). At only 40 and 50 Hz, torque was ∼30% less at day 176 than day 80 (*P* = 0.001–0.003) (Figure 4a). No torque values differed between day 40 and 120 or day 176, nor between day 176 and 120. Therefore, overall, submaximal torque values tended to increase at day 80, then decrease back toward the day 40 values at day 120 and 176. There was no effect of group on the torque–frequency relationship (*F*(1, 16) = 4.403, *P* = 0.052), indicating no differences between control and VCD.

There was an effect of time on *F*_50_ (*F*(2.109, 33.745) = 9.481, *P* < 0.001, η_p_^2^ = 0.372). Specifically, day 80 had a 13–16% lower *F*_50_ than days 40, 120 and 176 (*P* < 0.001–0.035) (Figure 4b). *F*_50_ at other time points did not differ from each other (*P* = 0.779–1.000). There was no effect of group on *F*_50_ (*F*(1, 16) = 0.241, *P* = 0.630), indicating no differences between control and VCD.

For the *n* slope coefficient, there was an effect of time (*F*(2.546, 40.731) = 4.870, *P* = 0.008, η_p_^2^ = 0.233). Specifically, day 80 had an 18% smaller slope coefficient than days 120 and 176 (*P* = 0.007–0.011). The slope coefficient at other time points did not differ from each other (*P* = 0.062–1.000). There was no effect of group on the slope coefficient (*F*(1, 16) = 0.254, *P* = 0.621), indicating no differences between control and VCD.

### 3.4 Differences in the torque–velocity–power relationship between control and VCD and across time

**FIGURE 5:**
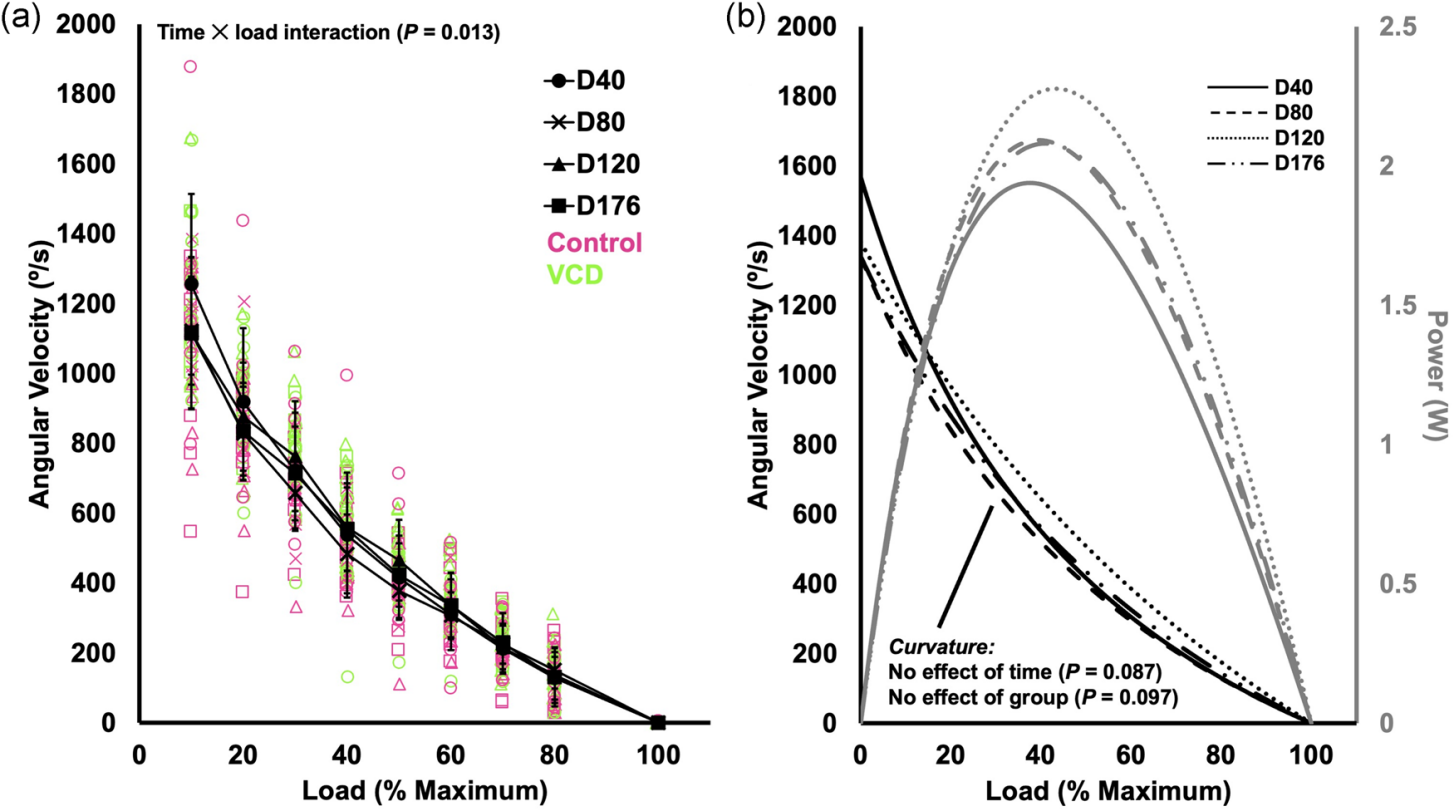
(a) The torque–angular velocity relationship at each time point (day 40, 80, 120 and 176) with control (*n* = 8) and VCD (*n* = 10) grouped, as there was no effect of group. Velocity at each load differed from all other loads within each time point (*P* < 0.05), but no time points differed from each other in angular velocity at any loads (*P* > 0.05). Data are presented as means ± standard deviation. (b) The fitted torque–angular velocity curves (black lines) that were used to determine maximum shortening velocity and power (grey lines). There were no effects of time or group on the curvature of the torque–velocity curve.

There was a time × load interaction for angular velocity (*F*(4.217, 67.467) = 3.342, *P* = 0.013, η_p_^2^ = 0.173); however, pairwise comparisons only showed differences in angular velocity between all loads (*P* < 0.001–0.002), while no time points differed between each other for any loads (*P* = 0.067–1.000) (Figure 5a). There was no effect of group on the torque–angular velocity relationship (*F*(1, 16) = 2.422, *P* = 0.139), indicating no differences between control and VCD.

For curvature (*a*/100 Hz torque) of the torque–angular velocity relationship, there were no effects of time (*F*(1.424, 22.787) = 2.952, *P* = 0.087) or group (*F*(1, 16) = 3.109, *P* = 0.097), indicating no differences across time points or between control and VCD (Figure 5b).

**FIGURE 6:**
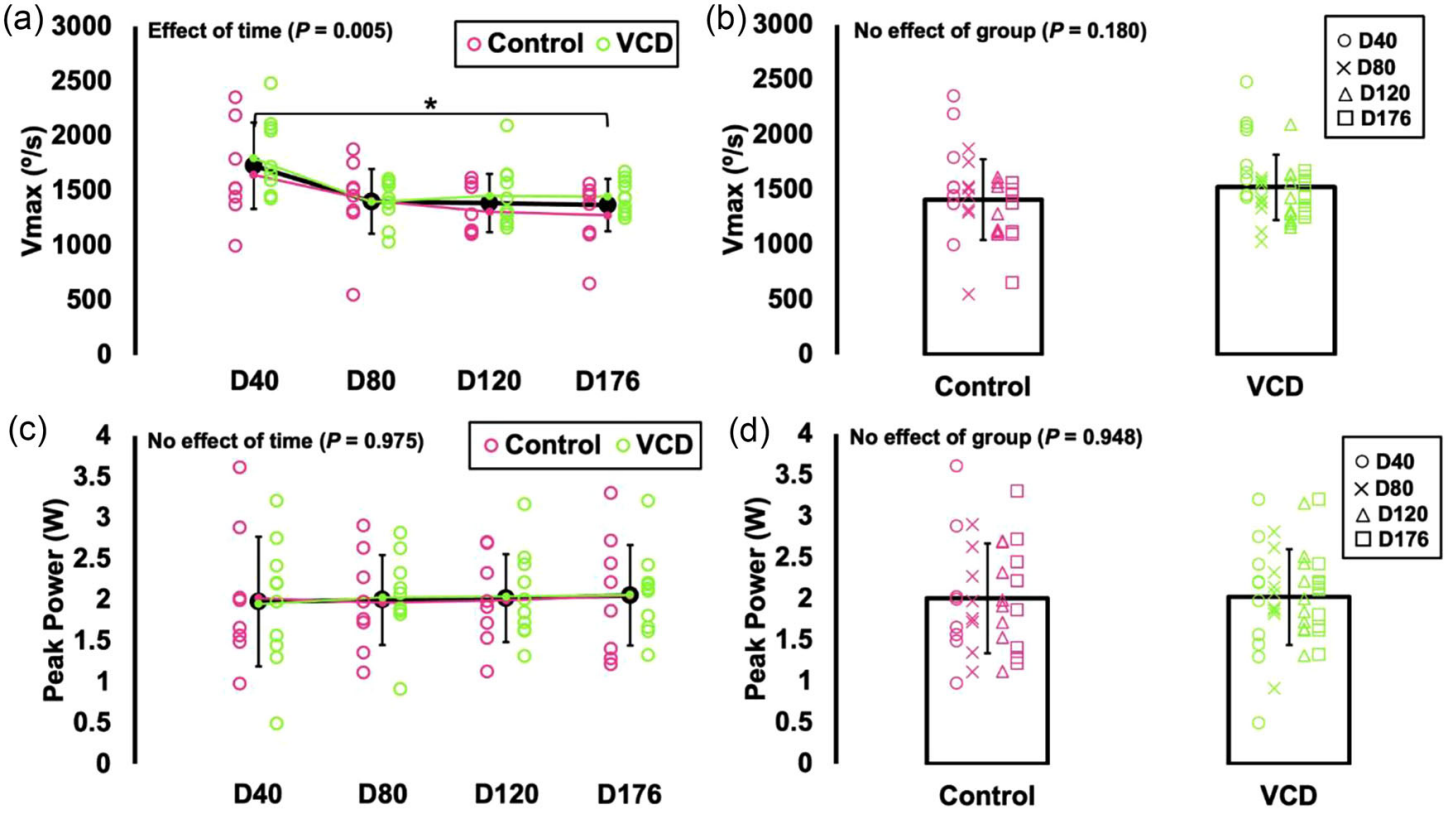
Differences in maximum shortening velocity (*V*_max_) (a, b) and peak isotonic power (c, d) across time (day 40, 80, 120 and 176) and between control (*n* = 8) and VCD (*n* = 10). Effects of time (control and VCD grouped) and effects of group (all time points grouped) are displayed because there was no time × group interaction for any measure. *Difference between indicated points (*P* < 0.05). Data are presented as means ± standard deviation.

For *V*_max_, there was an effect of time (*F*(2.093, 33.496) = 6.115, *P* = 0.005, η_p_^2^ = 0.277) with *V*_max_ being 21% slower at day 176 than day 40 (*P* = 0.011) (Figure 6a). There was no effect of group on *V*_max_ (*F*(1, 16) = 1.968, *P* = 0.180), indicating no differences between control and VCD (Figure 6b).

For peak power, there were no effects of time (*F*(2.612, 41.786) = 0.052, *P* = 0.975) or group (*F*(1, 16) = 0.004, *P* = 0.948), indicating no differences across time points or between control and VCD (Figure 6c,d). There were also no effects of time or group on torque at peak power (time: *F*(2.708, 43.333) = 2.777, *P* = 0.058; group: *F*(1, 16) = 2.497, *P* = 0.134) or velocity at peak power (time: *F*(2.079, 33.263) = 2.428, *P* = 0.102; group: *F*(1, 16) = 2.405, *P* = 0.140).

### 3.5 Differences in repetitions to failure between control and VCD and across time

**FIGURE 7:**
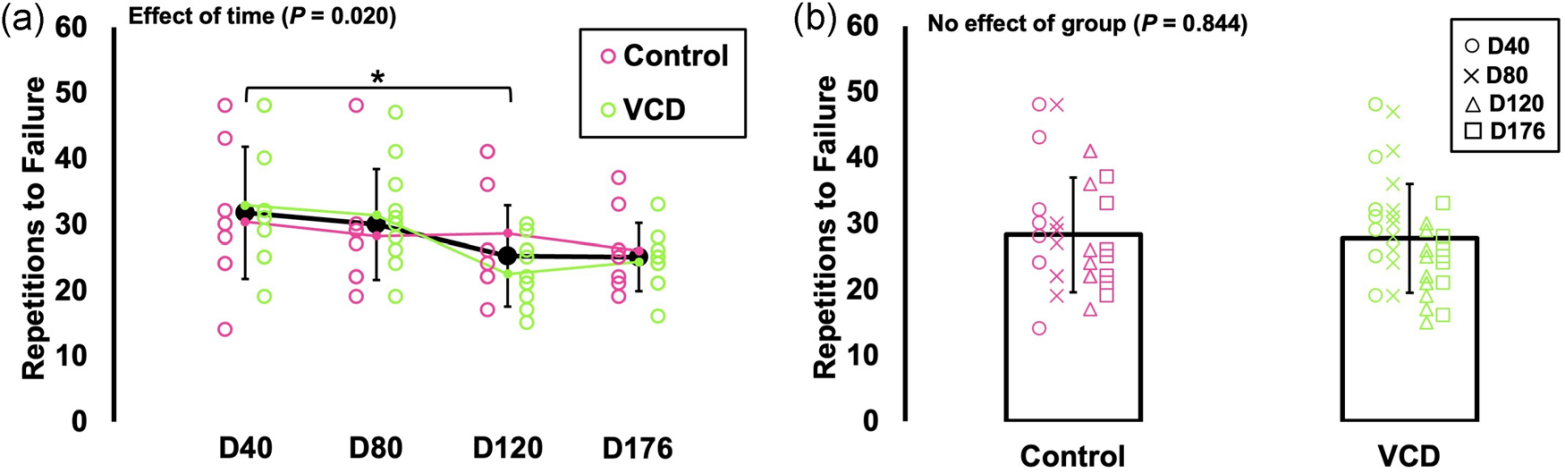
Differences in repetitions to failure across time (day 40, 80, 120 and 176) and between control (*n* = 8) and VCD (*n* = 10). Effects of time (control and VCD grouped) (a) and effects of group (all time points grouped) (b) are displayed because there was no time × group interaction for any measure. *Difference between indicated points (*P* < 0.05). Data are presented as mean ± standard deviation.

There was an effect of time on repetitions to failure (*F*(2.204, 35.262) = 4.219, *P* = 0.020, η_p_^2^ = 0.209), with mice having 19% fewer repetitions to failure at day 120 compared to day 40 (*P* = 0.041) (Figure 7a). There was no effect of group on repetitions to failure (*F*(1, 16) = 0.040, *P* = 0.844), indicating no difference between control and VCD (Figure 7b).

### 3.6 Recovery of twitch characteristics following the fatigue task

**FIGURE 8:**
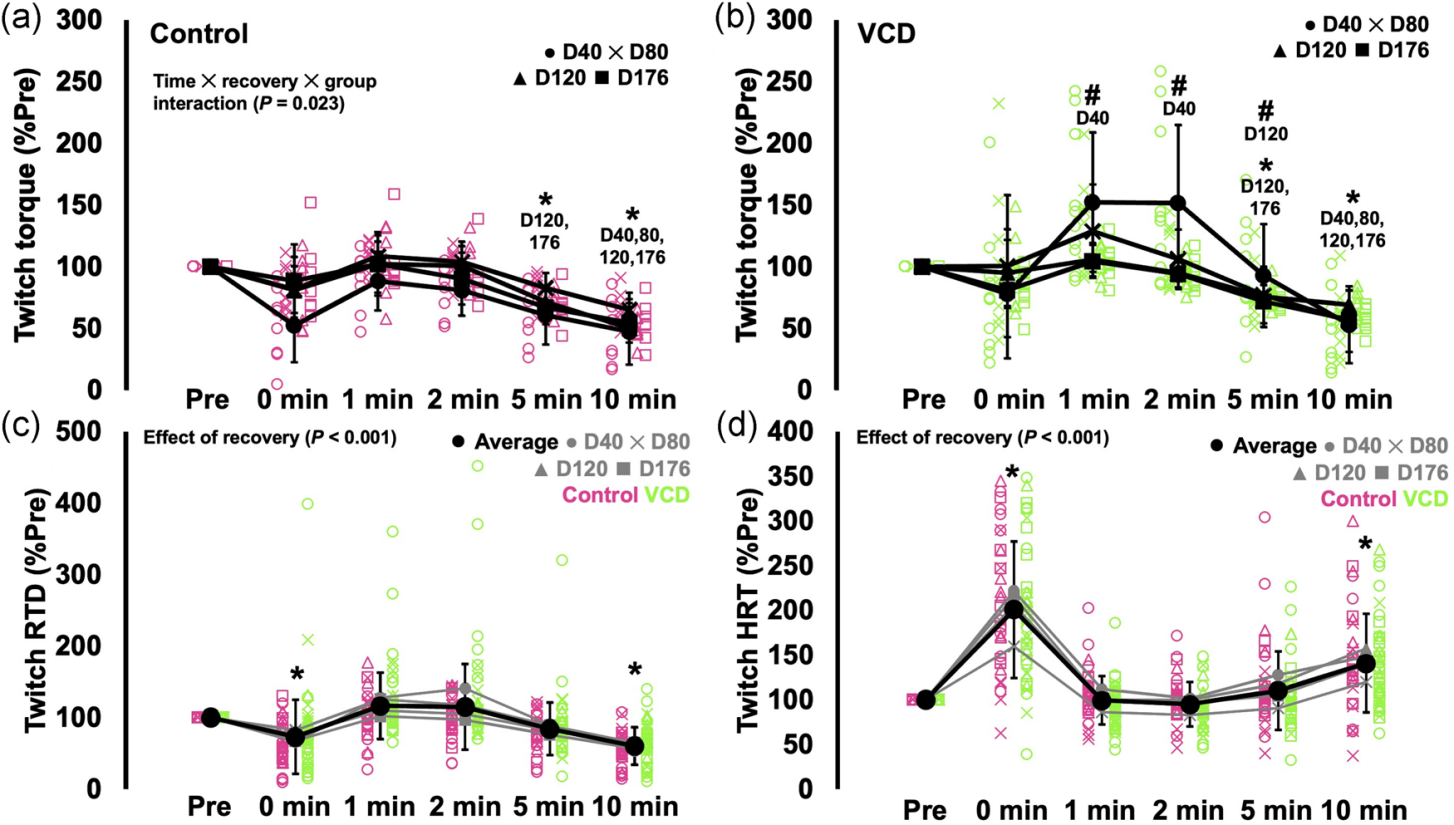
Recovery of peak twitch torque in control (a) and VCD (b) mice, twitch rate of torque development (RTD) (c), and twitch half‐relaxation time (HRT) (d) from pre‐fatigue up to 10 min following termination of the fatigue task. For (c, d), time points (day 40, 80, 120 and 176) and group (control (*n* = 8) and VCD (*n* = 10)) are grouped because there were no interactions of any factors, with only main effects of recovery. *Difference from pre (*P* < 0.05). #Difference between control and VCD (a, b). Data are presented as means ± standard deviation.

For peak twitch torque, there was a time × recovery × group interaction (*F*(4.238, 59.335) = 2.992, *P* = 0.023, η_p_^2^ = 0.176). For control and VCD at all time points, there was initially no drop in twitch torque immediately following the fatigue task compared to baseline (*P* = 0.188–1.00) (Figure 8a,b). At days 40 and 80, twitch torque was depressed by 35–53% at 10 min following the fatigue task (*P* = 0.006–0.016), and at days 120 and 176 by 29–50% at 5 (*P* < 0.001) and 10 min (*P* < 0.001) following the fatigue task (Figure 8a). Similarly, for VCD, twitch torque was 45–48% depressed at 10 min for days 40 and 80 (*P* < 0.001–0.008), and 25–53% depressed at 5 (*P* < 0.001) and 10 min (*P* < 0.001) following the fatigue task. Additionally, twitch torque relative to baseline was 62–68% greater 1 and 2 min following the fatigue task at D40 in VCD compared to control (*P* = 0.021), and 19% greater 5 min following the fatigue task at D120 in VCD compared to control (*P* = 0.008) (Figure 8b).

Twitch RTD did not have effects of time (*F*(1.323, 18.528) = 0.724, *P* = 0.443) or group (*F*(1, 14) = 2.539, *P* = 0.133) during the recovery period, indicating similar recovery at each time point and between control and VCD. There was an effect of recovery on twitch RTD (*F*(2.140, 29.965) = 28.708, *P* < 0.001, η_p_^2^ = 0.672), with twitch RTD being lower than baseline immediately (−26%, *P* = 0.037) and 10 min (−39%, *P* < 0.001) following the fatigue task (Figure 8c). Therefore, twitch RTD did not fully recovery by 10 min for either group at any time point.

Twitch HRT also did not have effects of time (*F*(1.906, 26.683) = 2.403, *P* = 0.112) or group (*F*(1, 14) = 0.686, *P* = 0.422) during the recovery period, indicating similar recovery at each time point and between control and VCD. There was an effect of recovery on twitch HRT (*F*(2.358, 33.007) = 107.622, *P* < 0.001, η_p_^2^ = 0.885) such that twitch HRT was 100% slower than baseline immediately following the fatigue task (*P* < 0.001) and 43% slower than baseline 10 min following the fatigue task (*P* < 0.001) (Figure 8d). Therefore, twitch HRT also did not fully recover by 10 min for either group at any time point.

### 3.7 Recovery of tetanic torque, tetanic RTD, 10 Hz torque and the 10:100 Hz torque ratio following the fatigue task

**FIGURE 9:**
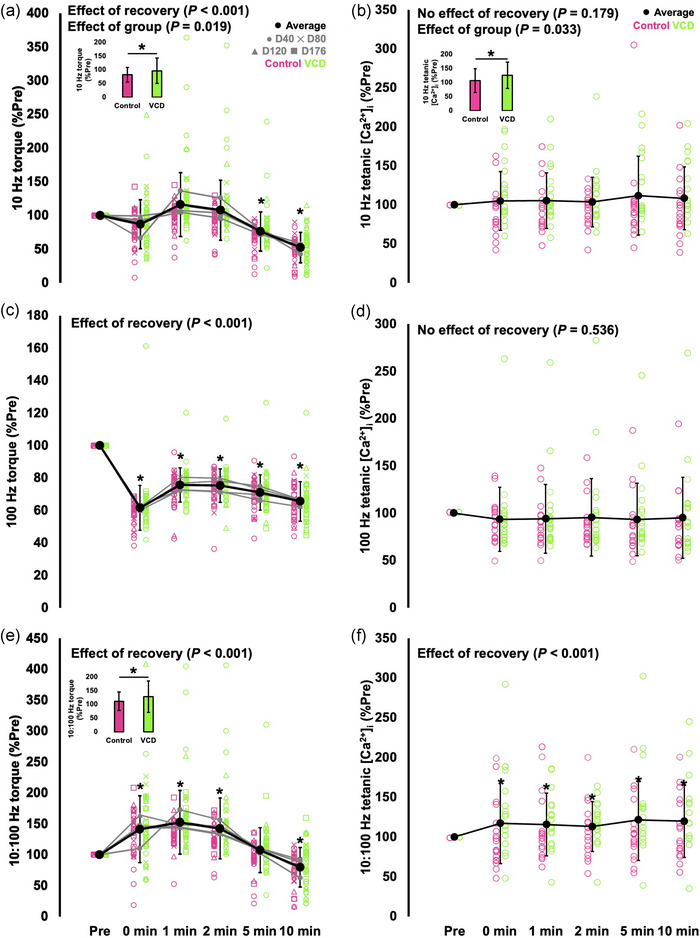
Recovery of 10 Hz torque (a), 10 Hz tetanic [Ca^2+^]_i_ (Fura‐2 ratio) (b), 100 Hz torque (c), 100 Hz tetanic [Ca^2+^]_i_ (Fura‐2 ratio) (d), 10:100 Hz torque (e) and 10:100 Hz [Ca^2+^]_i_ (Fura‐2 ratio) (f) following the fatigue task. The insets in (a, b, e) show effects of group with all time points combined. (a, c, e) are from *n* = 8 control and *n* = 10 VCD mice. (b, d, f) are from *n* = 35–39 fibres from *n* = 6 control and *n* = 8 VCD mice. *Significant difference between points (*P* < 0.05). Data are presented as means ± standard deviation.

Ten Hertz torque during the recovery period did not have an effect of time (*F*(1.653, 23.142) = 0.095, *P* = 0.876) but had an effect of group (*F*(1, 14) = 6.962, *P* = 0.019, η_p_^2^ = 0.757), indicating VCD and control differed in how 10 Hz torque recovered from the fatigue task. Specifically, VCD mice (97% of baseline) were overall relatively closer to pre‐fatigue levels than controls (82% of baseline) during the recovery period. There was also an effect of recovery (*F*(1.953, 27.345) = 43.584, *P* < 0.001, η_p_^2^ = 0.757). Specifically, 10 Hz torque was 24–48% lower than pre‐fatigue levels at 5 (*P* < 0.001) and 10 min (*P* < 0.001) following the fatigue task, indicating incomplete recovery (Figure 9a).

One hundred Hertz torque did not have effects of time (*F*(1.376, 19.270) = 1.405, *P* = 0.262) or group (*F*(1, 14) = 0.233, *P* = 0.637) during the recovery period, indicating similar recovery at each time point and between control and VCD. There was an effect of recovery on 100 Hz torque (*F*(2.229, 31.203) = 136.683, *P* < 0.001, η_p_^2^ = 0.907) such that 100 Hz torque experienced a 38% drop immediately following the fatigue task, and remained lower than baseline throughout the recovery period such that it was 34% lower than baseline by 10 min (all comparisons to baseline *P* < 0.001) (Figure 9c).

For the 10:100 Hz ratio during the recovery period, there was an effect of time (*F*(1.855, 25.973) = 0.471, *P* = 0.616) and an effect of group (*F*(1, 14) = 11.211, *P* = 0.005, η_p_^2^ = 0.445), indicating VCD and control differed in how the 10:100 Hz ratio recovered from the fatigue task. Specifically, VCD mice (128% of baseline) were overall relatively more above baseline than controls (110% of baseline) during the recovery period. There was also an effect of recovery (*F*(2.130, 29.816) = 36.358, *P* < 0.001, η_p_^2^ = 0.722). Specifically, the 10:100 Hz ratio was 40–41% greater than baseline immediately (*P* = 0.002) and 1 (*P* < 0.001) and 2 min (*P* < 0.001) following the fatigue task, then 20% lower than baseline (*P* < 0.001) 10 min following the fatigue task (Figure 9e).

For tetanic RTD, there were no effects of time (*F*(1.478, 20.692) = 1.180, *P* = 0.313) or group (*F*(1, 14) = 0.358, *P* = 0.559) during the recovery period, indicating similar recovery at each time point and between control and VCD. There was an effect of recovery on tetanic RTD (*F*(2.511, 35.157) = 24.639, *P* < 0.001, η_p_^2^ = 0.638), with tetanic RTD being 17% lower than baseline immediately following the fatigue task (*P* = 0.004) and 15–36% lower than baseline 2–10 min following the fatigue task (*P* < 0.001–0.011), indicating incomplete recovery.

### 3.8 Differences in the Ca^2+^–frequency relationship between control and VCD at day 176

**FIGURE 10:**
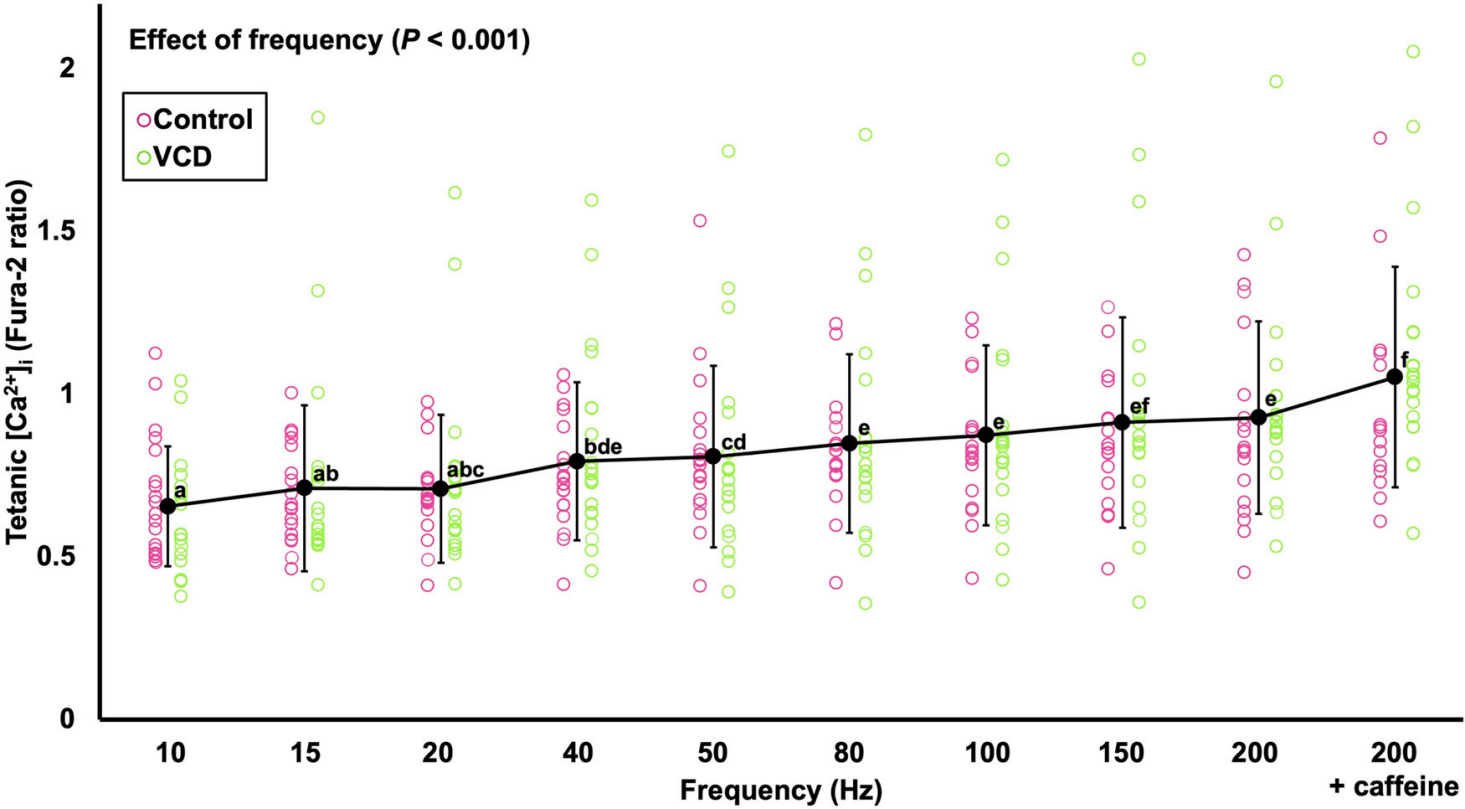
The mean tetanic Ca^2+^ concentration ([Ca^2+^])–frequency relationship with control and VCD grouped (*n* = 35–39 fibres from *n* = 6 control and *n* = 8 VCD mice) because there was no effect of group. Different letters indicate a significant difference between points (*P* < 0.05). Data are presented as means ± standard deviation.

There was no effect of group (*F*(1, 31) = 1.357, *P* = 0.253) on tetanic [Ca^2+^]_i_, indicating no differences in SR Ca^2+^ release between control and VCD at any stimulation frequency. There was only an effect of frequency (*F*(3.208, 99.436) = 23.978, *P* < 0.001, η_p_^2^ = 0.436) which showed, as expected, that tetanic [Ca^2+^]_i_ tended to increase with increasing stimulation frequency (*P* < 0.001–0.029), with 200 Hz and caffeine producing the highest tetanic [Ca^2+^]_i_ (Figure 10).

### 3.9 Differences in the recovery of 10 and 100 Hz tetanic [Ca^2+^]_i_ following fatigue between VCD and control

**FIGURE A1:**
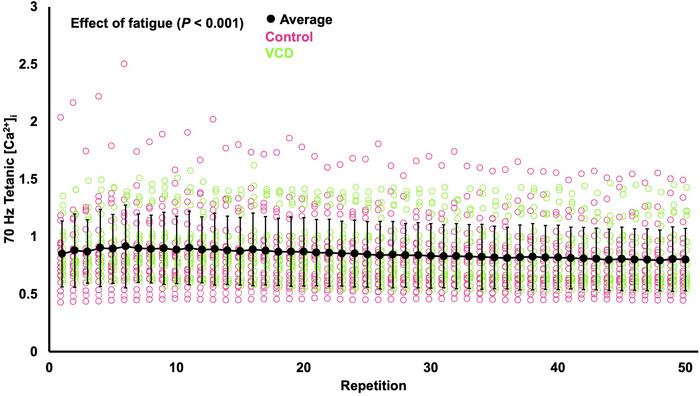
Mean 70 Hz tetanic [Ca^2+^]_i_ during the fatigue task (*n* = 39 fibres from *n* = 6 control and *n* = 8 VCD mice). Data are presented as means ± standard deviation with control and VCD data grouped together because there was no effect of group.

There was no effect of group on tetanic [Ca^2+^]_i_ during the fatigue task (*F*(1, 37) = 0.101, *P* = 0.752), indicating no differences in SR Ca^2+^ release during fatigue between control and VCD. There was, however, an effect of fatigue (*F*(3.069, 113.546) = 13.837, *P* < 0.001, η_p_^2^ = 0.272), with an overall drop in tetanic [Ca^2+^]_i_ of 13% from the highest (on average the 6th stimulation) to lowest (on average the 48th stimulation) points of the fatigue task (Figure A1).

There was no effect of recovery on 10 Hz tetanic [Ca^2+^]_i_ (*F*(3.200, 102.386) = 1.654, *P* = 0.179), indicating no change throughout the recovery period (Figure 9b). However, there was an effect of group (*F*(1, 32) = 4.985, *P* = 0.033, η_p_^2^ = 0.135) that showed VCD mice (118% of baseline) had higher 10 Hz tetanic [Ca^2+^]_i_ relative to baseline throughout the recovery period than controls (97% of baseline) (Figure 9b).

There was no effect of recovery (*F*(1.787, 58.966) = 0.596, *P* = 0.536) or group (*F*(1, 33) = 0.843, *P* = 0.365) on 100 Hz tetanic [Ca^2+^]_i_, indicating no differences throughout the recovery period nor between groups (Figure 9d).

There was no effect of group (*F*(1, 32) = 1.849, *P* = 0.183), but there was an effect of recovery on 10:100 Hz tetanic [Ca^2+^]_i_ (*F*(1.867, 59.753) = 108.087, *P* < 0.001, η_p_^2^ = 0.772) such that the 10:100 Hz tetanic [Ca^2+^]_i_ was 99–121% elevated from baseline throughout the recovery period (all *P* < 0.001 compared to baseline) (Figure 9f).

## 4 DISCUSSION

The present study investigated changes in mechanical function of the mouse plantar flexors during and after VCD‐induced gradual ovarian failure, mimicking the perimenopausal (days 40 and 80) transition into early and late‐stage menopause (days 120 and 176). We hypothesized that VCD‐induced ovarian failure would cause a gradual reduction in strength and power production, and increase fatiguability. Surprisingly, we found that VCD mice were only weaker than controls for twitch torque when all time points were grouped together. VCD and control mice did not differ for any other measures. Instead, VCD and control mice experienced similar time course changes in mechanical function. As well, VCD and controls exhibited mostly similar recovery from the fatigue task, with the exception of twitch torque, 10 Hz torque and 10 Hz tetanic [Ca^2+^], in which VCD mice were greater relative to pre‐fatigue throughout the recovery period. Taken together, these results indicate that VCD‐induced gradual ovarian failure does not have large detriments on in vivo mechanical function but does cause minor changes during recovery from fatigue during low‐frequency stimulation.

Our values of torque as a function of frequency, angular velocity and power are within ranges reported previously for the mouse plantar flexors in vivo (Baltgalvis et al., 2012; Hamm et al., 2021; Song et al., 2019; Watanabe et al., 2021; Wenke et al., 2010). As well, our values of tetanic [Ca^2+^]_i_ are within ranges reported previously from mouse FDB intact fibres (Chin et al., 2014). Therefore, despite changes in mechanical function at the single fibre level (Hubbard et al., 2023; Mashouri et al., 2023) following ovarian failure, these impairments do not seem to scale to the joint level possibility owing to some compensatory factors (discussed below).

### 4.1 The time course of changes in mechanical function in control and VCD mice

The only measure for which there was an effect of group was peak twitch torque, with VCD mice producing 16% lower twitch torque than controls when all time points were grouped together (Figure 2b). For all other outcome measures, VCD mice did not differ from controls. There were, however, effects of time (i.e., control and VCD groups combined) for several outcome measures. Twitch HRT became 34% faster from day 40 to 176, twitch RTD became 15% faster from day 80 to 176, *V*_max_ decreased 21% from day 40 to 176, and repetitions to failure decreased 19% from day 40 to 120. Additionally, submaximal levels of torque (10–80 Hz stimulation) increased 23–60% from day 40 to 80, then decreased back to values similar to day 40 by 176 (Figure 4a). In addition to not differing between control and VCD mice, maximum isometric torque and peak power did not differ between any time points. Since control and VCD mice did not differ for any of these measures and exhibited a similar time course of changes (or lack thereof), our findings highlight that baseline in vivo muscle mechanical function is minimally impacted by changes in ovarian hormone production.

Our findings are similar to other studies investigating how ovarian hormones impact in vivo mouse muscle function. Eight weeks following removal of the ovaries, Watanabe et al. (2021) observed no differences in the plantar flexor torque–frequency relationship between OVX mice, controls and OVX mice that received 17β‐estradiol. Others have also observed no differences in maximum isometric dorsiflexion torque between control and OVX mice 8 weeks following removal of the ovaries (Kosir et al., 2015; Vang et al., 2021). Cabelka et al. (2019) also observed no difference in peak power of the plantar flexors in vivo between skmERαKO mice and wild‐type mice, and only observed a small (7%) difference in maximum isometric torque when sedentary and trained groups were combined to increase the sample size. Most notably, Hubal et al. (2005) observed not only no differences in in vivo maximum isometric torque of the plantar flexors between control and OVX mice, but also similar time course changes in maximum isometric torque between control and OVX mice during the 8 weeks following removal of the ovaries, similar to the present study's findings. Our results build on these previous studies by showing that measures of muscle function other than maximum isometric torque (*V*_max_, peak power, RTD, twitch HRT and fatigability) are not impacted at the joint level by a reduction in ovarian hormones, and for the first time followed the evolution of mechanical changes across the menopausal spectrum.

Our results are in contrast to previous studies that observed reduced strength and power in VCD and OVX mice and rats compared to controls in vitro at the cellular (Hubbard et al., 2023; Lai et al., 2016; Peyton et al., 2022; Tiidus, 2005; Wattanapermpool & Reiser, 1999) and whole muscle (Greising et al., 2011; Moran et al., 2006) levels. It seems these smaller‐scale effects in muscle do not scale to the joint level, possibly because inter‐muscle differences have also been observed for how muscle function changes with VCD‐induced gradual ovarian failure. For example, the fast‐type EDL is less affected by VCD than the more slow‐type soleus (Hubbard et al., 2023; Mashouri et al., 2023). Alongside the soleus, the plantar flexors (i.e., the muscle group tested in the present study) include the lateral and medial gastrocnemii and the plantaris, which predominantly comprise fast‐type fibres (Augusto et al., 2004; Gavin et al., 2006; Sher & Cardasis, 1976). Therefore, the preservation of muscle mechanical function at the joint level following VCD‐induced gradual ovarian failure may represent a lack of effect on the other plantar flexor muscles masking the larger effect on the soleus.

VCD works through the gradual, selective destruction of primary and primordial cells in the ovaries, which leads to a state of ovarian failure, mimicking the natural trajectory of menopause. A benefit of using this model is the maintenance of androgen production as the ovarian theca cells remain intact, which can later be converted into estrogens in peripheral tissues via the aromatase enzyme (Magoffin, 2005; Mayer et al., 2004) as well as having direct effects on muscle. Thus, an important differentiating factor for the VCD model is that it allows for the examination of both gradual estrogen decline and continued androgen production, and how the relative proportion of these hormones affects muscle function. Androgens play an important role in maintaining muscle mass and strength (Mayer et al., 2004), and thus the continued production of androgens in the VCD model likely contributed to the lack of difference between the control and VCD groups that was observed for most of our measures. The VCD model allows for assessments of gradual hormonal changes, mimicking natural menopause, and thus proves to be a more physiologically relevant model compared to the OVX model (Brooks et al., 2016; Hoyer et al., 2001). By using the VCD model and conducting longitudinal assessments in vivo across the perimenopausal and menopausal phases, we have presented arguably the most physiologically relevant results regarding how changes in circulating ovarian hormones during menopause impact muscle mechanical function.

In studies on humans, menopause has been shown to impact skeletal muscle strength. While it has been shown that ageing contributes to loss of muscle strength in menopausal women, the loss of estradiol throughout menopause has been identified as a key contributor, as administration of estrogen replacement therapy has been shown to mitigate strength loss (Greising et al., 2009; Maltais et al., 2009; Sipilä et al., 2001; Sirola & Rikkonen, 2005). Differences observed between our results compared to the human literature on menopause may be explained by our final time point of 8 weeks post‐menopause (i.e., day 176) possibly not being far enough into menopause to show detriments to muscle mechanical performance. As well, and most likely, in the studies on humans post‐menopause, it is possible that ageing combined with reduced circulating 17β‐estradiol contributed more to the observations of strength loss. By contrast, ageing was unlikely to impact our mice, as our day 176 for mice corresponds to ∼48 years old in humans (Wang et al., 2020).

### 4.2 Differences in impairment and recovery from fatigue between control and VCD

Time to task failure was not different between groups, and maximal torque production exhibited a similar response following the fatigue task across both groups and all time points, demonstrating no effect of VCD‐induced gradual ovarian failure on the recovery of torque following fatigue during the perimenopausal or menopausal phases. There was an initial 39% drop in 100 Hz torque immediately following the fatigue task, and still a 34% reduction compared to pre‐fatigue values by 10 min. Similarly, tetanic RTD experienced an initial 17% drop immediately following the fatigue task, then still a 32% reduction compared to pre‐fatigue values by 10 min. Cabelka et al. (2019) similarly observed still an ∼35% reduction in maximum force of the isolated soleus 20 min following an isometric fatigue task, but only in skmERαKO mice, while controls were 90% recovered at 5 min. Cabelka et al. (2019)’s differing findings compared to ours regarding the effect of ovarian hormones on recovery following fatigue can likely be attributed to a lack of scaling from the whole‐muscle to the in vivo joint level as discussed above. Our findings better align with previous observations in humans, in which time to task failure during a voluntary isometric fatigue task of the thumb adductors did not differ between menopausal women (∼70 years of age) on hormone replacement therapy and not on hormone replacement therapy (Cheng et al., 2003).

Unlike with the 100 Hz contractions, for submaximal levels of force production, we observed some differences in recovery from the fatigue task between groups and across time points. While twitch RTD and twitch HRT showed similar fatigability and recovery between groups and across time points (a 26% reduction in twitch RTD and a 100% increase in twitch HRT immediately following task failure, and still ∼40% deficits in those measures by 10 min), recovery of twitch torque differed depending on the group and the time point. Twitch torque was not significantly reduced immediately following the fatigue task for either group at any time point, but tended to decrease by 5 min, and did not recover by 10 min (Figure 8a,b). Where control and VCD mice differed was at the times 1–5 min following task failure. At day 40, VCD mice exhibited 62–68% greater twitch torque relative to pre‐fatigue than control mice 1 and 2 min following task failure. Similarly, at day 120, VCD mice exhibited 19% greater twitch torque relative to pre‐fatigue than control mice 5 min following task failure. Ten Hertz torque also exhibited a slightly different recovery profile between control and VCD mice. While both groups showed no significant reduction in 10 Hz torque immediately following task failure and a 48% reduction compared to baseline at 10 min, VCD mice produced 15% greater 10 Hz torque relative to pre‐fatigue than controls across the whole recovery period (inset in Figure 9a). Greater 10 Hz torque across the recovery period also corresponded to a 21% greater 10 Hz tetanic [Ca^2+^]_i_ relative to pre‐fatigue in VCD than control intact single fibres across the recovery period (inset in Figure 9b). Lower stimulation frequencies (i.e., 1 and 10 Hz) are more influenced by Ca^2+^ handling (Ca^2+^ release and sensitivity) than higher stimulation frequencies (i.e., 100 Hz). Therefore, these perplexing larger increases in twitch torque and 10 Hz torque in VCD mice around the middle of the recovery period are likely associated with the greater Ca^2+^ observed at those recovery time points.

The apparent increases in low‐frequency torque and 10 Hz tetanic [Ca^2+^]_i_ throughout recovery may be indicative of post‐tetanic potentiation, which is marked by increased force production at low‐frequency stimulation, sometimes masking the force loss that normally accompanies fatigue (MacIntosh, 2010; Vandenboom et al., 2013). Full tetanic stimulation results in the maximal release of Ca^2+^ from the SR, which alongside calmodulin then activates the enzyme myosin light chain kinase (MLCK). MLCK phosphorylates the myosin regulatory light chain, which (1) brings myosin into closer proximity to actin and (2) increases Ca^2+^ sensitivity, increasing the probability of crossbridge attachment (Vandenboom et al., 2013). These mechanisms result in a greater number of attached crossbridges and thus potentiation of force when a low‐frequency stimulation is delivered during the first few minutes following maximal activation. An increased number of crossbridge attachments (a feature of potentiation as described above) during recovery in VCD mice may have been evident in our results as well, as there was a trend toward a time × recovery × group interaction (*P* = 0.054) for twitch RTD, with VCD mice at day 40 exhibiting noticeably greater twitch RTD 1–2 min into the recovery period (see green data points in Figure 8c).

It seems that at 1–2 min into the recovery period, VCD mice experienced a greater potentiation effect than control mice, after which potentiation subsided, with the force loss associated with muscle fatigue overtaking potentiation at 5 and 10 min. This greater potentiation effect in VCD mice may be a result of greater SR Ca^2+^ release, as suggested by the greater 10 Hz tetanic [Ca^2+^]_i_ across the recovery period in VCD mice (inset in Figure 9b). If potentiation is caused by greater Ca^2+^ sensitivity, this elevated SR Ca^2+^ release in VCD mice would more fully saturate Ca^2+^ binding sites on troponin C, enhancing the effect of post‐tetanic potentiation. Ca^2+^ binding to myosin also increases the mobility of the S1 heads, which contributes to bringing myosin into closer proximity to actin (Podlubnaya et al., 1999). Fibre type may also play a role here. Mashouri et al. (2023) and Hubbard et al. (2023) recently showed that VCD‐induced ovarian failure is associated with a shift toward a greater proportion of MHC II fibres (e.g., fast twitch fibres) in the mouse soleus. Fast twitch muscle has a higher activity of MLCK and lower activity of phosphatase C (which dephosphorylates the myosin regulatory light chain), and correspondingly exhibits greater twitch potentiation than slow twitch muscle (Moore & Stull, 1984). Therefore, the greater potentiation effect observed in VCD compared to control mice during the recovery period may be due to a VCD‐induced shift toward more fast twitch fibres in the soleus (i.e., one of the muscles contributing to plantar flexion torque in the present study). With that said, a faster twitch HRT may be indicative of more fast twitch fibres, and we did not observe differences in HRT (which, being a measure of the speed of twitch relaxation, provides insight on the predominant fibre type for a muscle group) between control and VCD mice. As well, since we did not quantify whole muscle fibre type proportion in the present study, we can only speculate on fibre type changes/differences. Overall, our results on twitch and 10 Hz torque indicate that ovarian failure might modulate the interaction between fatigue and post‐tetanic potentiation during recovery following an isometric fatigue task (Place et al., 2005). Further research is warranted to better elucidate mechanisms contributing to this greater potentiation effect in VCD compared to control mice (e.g., differences in activity of MLCK and phosphoprotein phosphatase C, fibre type differences).

### 4.3 Conclusion

The VCD model is known for its better representation of the natural progression of human menopause compared to the OVX model as previously discussed by Brooks et al. (2016). Additionally, assessment of muscle mechanical function in vivo at the joint level bears better translatability to everyday movement than reduced preparations (i.e., whole muscle and single fibre experiments). Thus, by performing longitudinal in vivo assessments up to 25 weeks following VCD injection, we have provided arguably the most physiologically relevant assessment of changes in muscle mechanical function during the development of menopause. While we noted minimal differences in pre‐fatigue measures of muscle mechanical function between control and VCD mice, differences in potentiation during recovery from fatigue should be investigated further in future studies. Owing to the potential role of estrogen receptors in regulating transcriptomic alterations of muscle with age (Yoh et al., 2023), future studies should explore why findings differ across models of menopause in the context of human ageing, and importantly the concomitant effects of both ageing and reduced circulating sex hormones on muscle mechanical function.

## AUTHOR CONTRIBUTIONS

Conceptualization: Benjamin E. Dalton, Avery Hinks, Parastoo Mashouri and Geoffrey A. Power. Data curation: Benjamin E. Dalton, Avery Hinks, Parastoo Mashouri and Geoffrey A. Power. Formal analysis: Benjamin E. Dalton, Avery Hinks, Parastoo Mashouri and Geoffrey A. Power. Funding acquisition: Geoffrey A. Power. Investigation: Benjamin E. Dalton, Avery Hinks and Luke D. Flewwelling. Methodology: Benjamin E. Dalton, Avery Hinks, Luke D. Flewwelling, Arthur J. Cheng, William Glen Pyle and Geoffrey A. Power. Supervision: Geoffrey A. Power. Writing—original draft: Benjamin E. Dalton, Avery Hinks, Parastoo Mashouri and Geoffrey A. Power. Writing—review & editing: Benjamin E. Dalton, Avery Hinks, Parastoo Mashouri, Luke D. Flewwelling, Arthur J. Cheng, William Glen Pyle and Geoffrey A. Power. All authors have read and approved the final version of this manuscript and agree to be accountable for all aspects of the work in ensuring that questions related to the accuracy or integrity of any part of the work are appropriately investigated and resolved. All persons designated as authors qualify for authorship, and all those who qualify for authorship are listed.

## CONFLICT OF INTEREST

The authors declare no conflicts of interest.

## Data Availability

Supporting data are available upon request.
